# Hypoxia-ameliorated photothermal manganese dioxide nanoplatform for reversing doxorubicin resistance

**DOI:** 10.3389/fphar.2023.1133011

**Published:** 2023-02-24

**Authors:** Zhenzhen Chen, Zhihong Liu, Qian Zhang, Sheng Huang, Zaizhong Zhang, Xianquan Feng, Lingjun Zeng, Ding Lin, Lie Wang, Hongtao Song

**Affiliations:** ^1^ Department of General Surgery, 900TH Hospital of Joint Logistics Support Force, Fuzhou, China; ^2^ Department of Pharmacy, 900TH Hospital of Joint Logistics Support Force, Fuzhou, China; ^3^ College of Pharmacy, Fujian Medical University, Fuzhou, China; ^4^ Department of Pharmacy, Jiaxing Maternal and Child Healthcare Hospital, Affiliated Hospital of Jiaxing University, Jiaxing, China

**Keywords:** drug resistance, hypoxia, photothermal-manganese dioxide, doxorubicin, tumor microenvironment response

## Abstract

Drug resistance is a huge hurdle in tumor therapy. Tumor hypoxia contributes to chemotherapy resistance by inducing the hypoxia-inducible factor-1α (HIF-1α) pathway. To reduce tumor hypoxia, novel approaches have been devised, providing significant importance to reverse therapeutic resistance and improve the effectiveness of antitumor therapies. Herein, the nanosystem of bovine serum albumin (BSA)-templated manganese dioxide (MnO_2_) nanoparticles (BSA/MnO_2_ NPs) loaded with doxorubicin (DOX) (DOX-BSA/MnO_2_ NPs) developed in our previous report was further explored for their physicochemical properties and capacity to reverse DOX resistance because of their excellent photothermal and tumor microenvironment (TME) response effects. The DOX-BSA/MnO_2_ NPs showed good biocompatibility and hemocompatibility. Meanwhile, DOX-BSA/MnO_2_ NPs could greatly affect DOX pharmacokinetic properties, with prolonged circulation time and reduced cardiotoxicity, besides enhancing accumulation at tumor sites. DOX-BSA/MnO_2_ NPs can interact with H_2_O_2_ and H^+^ in TME to form oxygen and exhibit excellent photothermal effect to further alleviate hypoxia due to MnO_2_, reversing DOX resistance by down-regulating HIF-1α expression and significantly improving the antitumor efficiency in DOX-resistant human breast carcinoma cell line (MCF-7/ADR) tumor model. The hypoxia-ameliorated photothermal MnO_2_ platform is a promising strategy for revering DOX resistance.

## 1 Introduction

Currently, chemotherapy remains an important remedy for tumor treatment, while drug resistance is the primary obstacle that causes chemotherapy failure (Wei et al., 2021). Since tumor cells progress rapidly together with limited neovascularization, hypoxia is a characteristic of the tumor microenvironment (TME), defined by a low pH value, a high level of glutathione (GSH), and coupled with reactive oxygen species (including H_2_O_2_) in high concentration (Sharma et al., 2019; Wang et al., 2021). Experimental and clinical research has indicated that tumor hypoxia is the leading cause of chemotherapy resistance, which would trigger tumor recurrence, as well as metastasis, causing treatment failure, in addition to most tumor-based mortality (Rohwer and Cramer, 2011; Liu et al., 2021; Zhao et al., 2022). Moreover, hypoxia elevates chemotherapy drug resistance by inducing the hypoxia-inducible factor-1α (HIF-1α) pathway, which relates to angiogenesis, cancer growth, metastasis, metabolic reprogramming, as well as therapy resistance (Rankin and Giaccia, 2016; McAleese et al., 2021; Li H. et al., 2022). Therefore, HIF-1α is essential in reversing drug resistance (Jing et al., 2019; Xu et al., 2019).

Consequently, several techniques, including transporting oxygen (O_2_) into tumors through artificial blood substitutes (such as perfluorocarbon) or generating O_2_ *in situ* through O_2_-generating nano-delivery systems, overcome hypoxia in tumors that reduces the HIF-1α expression (Wang et al., 2018; Tang et al., 2021). Recently, developing TME-sensitive nanoagents is a promising approach to supply tremendous and sustained O_2_ production besides regulating the undesirable TME (Chen et al., 2016), therefore achieving better therapeutic outcomes against drug resistance. Until now, the supply of O_2_ according to the high reactivity of manganese dioxide (MnO_2_) nanoparticles (NPs) with H_2_O_2_ in a tumor acid environment is advantageous over other approaches to supplying O_2_ (Yu et al., 2019). Meanwhile, MnO_2_ NPs can be reduced to Mn^2+^ in glutathione (GSH) presence, leading to MnO_2_ NPs decomposition. The TME-responsive potential of MnO_2_ NPs would facilitate on-demand drug release. Importantly, kidneys can quickly eliminate harmless water-soluble Mn^2+^ ions from the body to prevent systemic buildup and chronic toxicity, providing great benefits for *in vivo* applications (Yang et al., 2017), in contrast to several other non-biodegradable inorganic nanomaterials (including mesoporous silica). Therefore, MnO_2_ NPs are excellent candidates for alleviating hypoxia in TME.

However, a single treatment to alleviate hypoxia shows limited effectiveness due to the complexity of TME. The dense extracellular matrix, increased interstitial fluid pressure, as well as irregular blood flow frequently act as physical barriers to the passage of medication or nanodrug through tumor blood microvessels into hypoxic areas (Chen et al., 2022). One of the non-invasive clinical treatment modalities is photothermal therapy (PTT), which relies on photothermal agents converting energy from light to heat, which can reinforce the extravasation of NPs treatment into the cancer interstitium. PTT can accelerate blood flow into tumors to help alleviate hypoxia, exhibiting competitive advantages in overcoming tumor drug resistance (Jiang et al., 2020). Moreover, PTT reinforces tumor vascular permeability, allowing antitumor drugs delivery to deep tumor tissues and elevating the sensitivity of tumor cells resistant to chemotherapeutic treatments by suppressing the efflux transporters expression, including multidrug resistance-associated protein 1 (MDR-1) (Souslova and Averill-Bates, 2004). Furthermore, developing a nanosystem that combines PTT with O_2_-generated NPs is a promising strategy for overcoming hypoxia in tumor sites and improving drug resistance, enhancing chemotherapy efficacy. Zhu et al. (2021) constructed Ru@MnO_2_ nanozymes, which showed both excellent photothermal conversion efficiency by Ru NPs and catalytic activity by MnO_2_ NPs to catalyze endogenous H_2_O_2_ for O_2_ production, reliving the hypoxic TME and enhancing chemotherapy efficiency. However, the complex synthetic steps, such as using nano-carriers to load O_2_-generating agents and PTAs, are time-consuming and probably lead to low drug loading and encapsulation efficiency, resulting in restricting the antitumor effect. Moreover, the use of too many reagents or excipients can reduce the biocompatibility of NPs, probably affecting their *in vivo* application. Therefore, developing a facile way to synthesize multifunctional NPs to improve tumor hypoxia to reverse drug resistance is of great significance.

**SCHEME 1:**
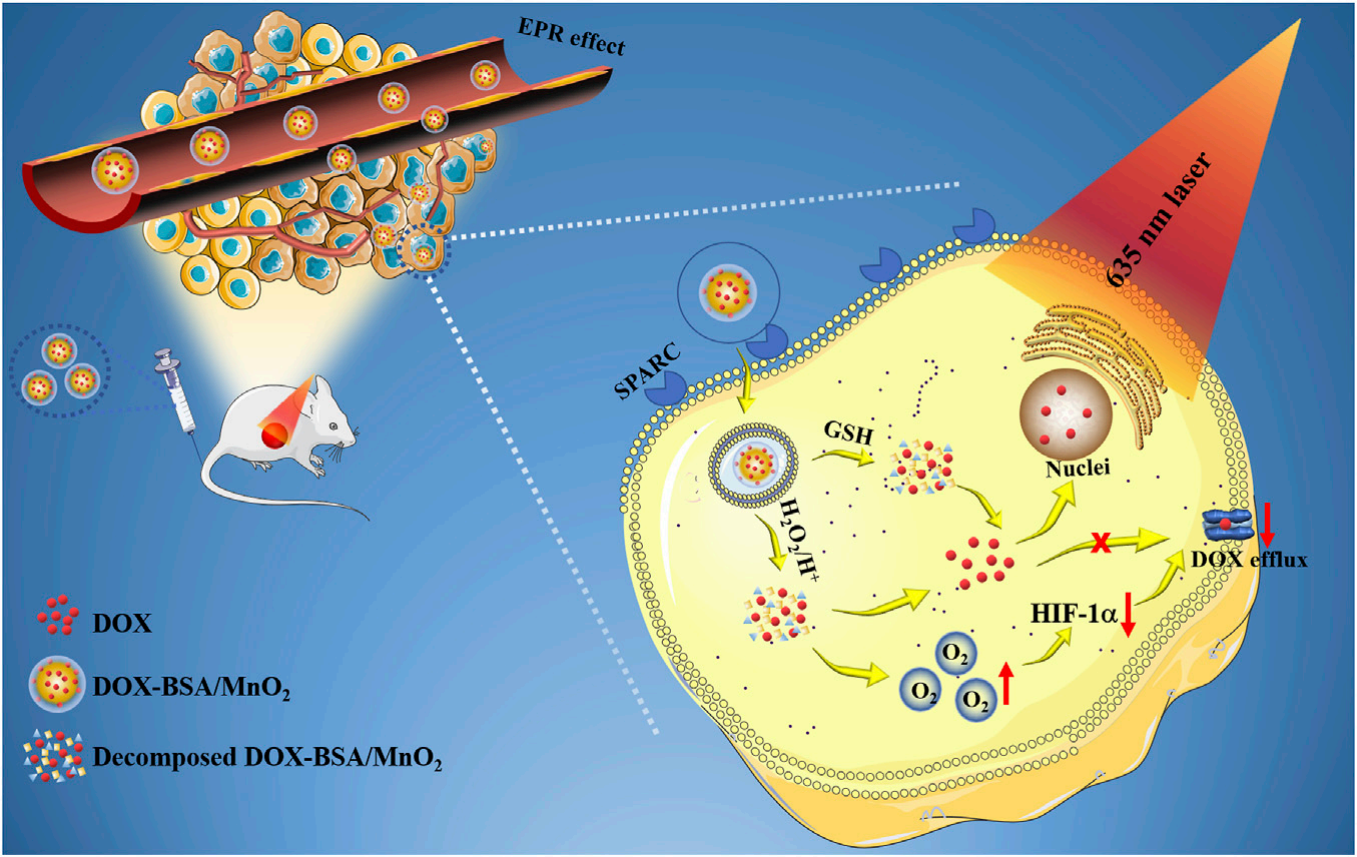
Diagrammatic representation of DOX-BSA/MnO_2_ NPs as a multifunctional platform to reverse DOX resistance (EPR: enhanced permeability and retention; DOX: Doxorubicin; MnO_2_: manganese dioxide; GSH: glutathione; HIF-1α: Hypoxia-inducible factor-1α).

In our previous study (Chen et al., 2022), bovine serum albumin (BSA) was employed as an organic reference to produce BSA/MnO_2_ NPs through biomimetic mineralization, a green, facile, and promising strategy to synthesize NPs for tumor therapy (Wang et al., 2020). The developed nanosystem has many advantages (Scheme 1): 1) BSA/MnO_2_ NPs showed increased biocompatibility, good stability, and high-water solubility due to the existence of BSA. 2) Importantly, by targeting the albumin-binding protein SPARC, which is upregulated in most cancerous cell cases, BSA can increase intratumoral accumulation *via* albumin receptor (gp60)-mediated transcytosis (Desai, 2007). 3) BSA/MnO_2_ NPs are multifunctional carriers that exhibit photothermal conversion ability under 635 nm laser irradiation and can catalyze O_2_ production at tumor sites. 4) Furthermore, the chemotherapy agent, doxorubicin (DOX), was loaded on BSA/MnO_2_ NPs by simply stirring in an aqueous solution to construct DOX-BSA/MnO_2_ NPs depending on MnO_2_ along with DOX coordination without further modification, and with high encapsulation efficiency (99.33%) and drug loading capacity (23.88%). 5) A 32.5 nm particle size DOX-BSA/MnO_2_ NPs prolonged blood circulation and efficient cancer buildup, enhanced permeability and retention (EPR) effect. Our previous research has proved the excellent photothermal effects, as well as the ability to relieve hypoxic TME of DOX-BSA/MnO_2_ NPs (Chen et al., 2022). It inspired us to explore its ability to reverse chemotherapy resistance. In particular, the combined BSA/MnO_2_ nanoplatform generated the thermal ability of O_2_ and was the first used to investigate the ability to reverse DOX resistance. Therefore, our objective was to explore the physicochemical properties and the capacity to reverse the drug resistance of DOX-BSA/MnO_2_ NPs.

## 2 Materials and methods

### 2.1 Materials

We purchased BSA from Sigma-Aldrich; KMnO_4_ from Sinopharm Chemical Reagent CO., Ltd. (Shanghai, China); DOX from Shanghai Aladdin Bio-Chem Technology CO., Ltd. (Shanghai, China); Daunorubicin (DNR) from Dalian Meilun Biotech Co., Ltd. (Dalian, China); Indocyanine green (ICG) from Tokyo Chemical Industry Co., Ltd. (Tokyo, Japan); Fetal bovine serum (FBS) from Elite (Marburg, Germany); Roswell Park Memorial Institute 1,640 (RPMI 1640) as well as 3-(4,5-dimethylthiazol-2-yl)-2,5-diphenyltetrazolium bromide (MTT) from Keygen Biotech Corp., Ltd. (Jiangsu, China). The Milli-Q system (Millipore, United States) purified the water. Other analytical grade chemicals were obtained from Sigma (St. Louis, United States) and utilized with no additional purification except as described elsewhere.

### 2.2 Methods

#### 2.2.1 Detecting the crystalline state of DOX

The freeze-dried samples of DOX, BSA/MnO_2_ NPs, physical mixture of DOX and BSA/MnO_2_ NPs, and DOX-BSA/MnO_2_ NPs analyzed DOX crystalline state through differential scanning calorimetry (DSC) besides X-ray diffraction (XRD) analyses. The detailed processes were as follows.

(1) DSC

DSC analysis was performed using Thermo Fisher DSC Xi250 (Thermo Fisher, United States). Briefly, 1 mg–5 mg of samples were accurately weighted and sealed inside an aluminum pan, and nitrogen was utilized as the purge gas at a flow rate of 40 mL/min. The heating rate was kept at 10°C/min from 25°C to 300°C. A reference aluminum pan was provided.

(2) XRD

XRD patterns of samples were obtained utilizing an X-ray diffractometer (XRD-6000, Shimadzu, Tokyo, Japan) with Cu Kα radiation (*λ* = 1.5418 Å, 40 kV, 40 mA), with 5°–90° at 2°/min speed as the scans-range (2θ) and 40 kV with a 40 mA tube current as the tube voltage.

#### 2.2.2 Hemolysis analysis

The hemolysis test was carried out according to the standard methods described in the Chinese Pharmacopoeia (2020). Briefly, fresh blood was collected from a New Zealand rabbit through a marginal ear vein*.* A 2% red blood cells (RBCs) suspension was obtained after fibrinogens were removed by slowly stirring using a glass rod and diluting with 0.9% NaCl solution. Then, 0.15 mL of DOX-BSA/MnO_2_ NPs at various doses (25 μg/mL, 50 μg/mL, 100 μg/mL, 200 μg/mL, and 500 μg/mL) and 1.10 mL of 0.9% NaCl were added into 1.25 mL of 2% RBCs suspension followed by 3 h, 37 °C water bath incubation. Purified water and 0.9% NaCl incubated with RBCs presented the positive and negative controls, respectively. The entire sample was then subjected to 300 rpm centrifugation for 10 min. Therefore, 200 µL of the supernatant was taken on a 96-well plate, and its absorbance (A) was assessed at a wavelength of 540 nm with a microplate reader (Model 680, Bio-Rad, United States). The hemolysis percentage was calculated as follows:

$$Hemolysis\;\left(\%\right)=\frac{A_{sample}-A_{0}}{A_{100}-A_{0}}\times 100\%$$

A_sample_, A_0_, and A_100_ refer to the sample absorbance values, negative control, and positive control groups, respectively.

#### 2.2.3 Cell culture

The murine and human breast carcinoma cell lines 4T1 and MCF-7, respectively, were acquired from the Chinese Academy of Sciences Shanghai Institute for Biological Sciences Cell Resource Center and incubated in RPMI 1640 and DMEM media. The anthracycline drug-resistant human breast carcinoma cell line, MCF-7/ADR, has been generously contributed by Professor Jianhua Xu, College of Pharmacy, Fujian Medical University, and cultivated in RPMI 1640 media. Moreover, the two media were enriched with 10% FBS, penicillin (100 U/mL), and streptomycin (100 mg/mL). Cell line culture was incubated at 37°C in a humidified setting with 5% CO_2_.

#### 2.2.4 Cytotoxicity analysis

In 96-well plates (1×10^4^ cells/well), MCF-7 and MCF-7/ADR cells were plated, followed by overnight incubation. Subsequently, the free DOX and DOX-BSA/MnO_2_ NPs at 0.001 μM–100 μM concentrations (at equivalent DOX concentration) were incubated for 24 h with the cells. MTT assay evaluated the cytotoxicity besides a microplate reader measured each well absorbance at 570 nm. GraphPad Prism 8.3.0 calculated the half-maximal inhibitory concentration (IC50) values.

#### 2.2.5 *In vitro* cellular uptake studies

MCF-7 and MCF-7/ADR cells were planted in laser confocal dishes (15 mm in diameter) with 1 × 10^5^ cells/well density and incubated for one night. Subsequently, the media was replaced with a new media enriched with 5 µM of DOX or DOX-BSA/MnO_2_ NPs (5 µM DOX-equivalent). Therefore, cells were incubated for 6 h, rinsed with PBS three times, and then preserved with 4% formaldehyde for 30 min. For another 10 min, cell nuclei were stained with 4′, 6-diamidino-2-phenylindole (DAPI), washed with PBS three times, and images were captured using confocal laser scanning fluorescence microscopy (CLSM, SP5, Leica, United States) to observe cell uptake and subcellular distribution.

For fluorescence intensity quantification, MCF-7 and MCF-7/ADR cells were planted into a 6-well plate at 3 × 10^5^ cells/well and incubated for one additional night, followed by replacing media with a fresh one containing either 5 µM of DOX or DOX-BSA/MnO_2_ NPs (5 µM DOX-equivalent). Cells were incubated for 6 h, rinsed three times with PBS, digested with 0.25% trypsin, and finally centrifuged to be harvested. Flow cytometry (FACSVerse, BD, US) measured the fluorescence intensity of DOX cellular uptake.

#### 2.2.6 Animal studies

Sprague-Dawley (SD) rats (180–220 g), female BALB/c mice (18–22 g), as well as BALB/c nude mice (18–22 g) were acquired from the Silaike Experimental Animal Limited Liability Company (Shanghai, China). The entire *in vivo* experiments followed the recommendations of the Institutional Animal Care and Use Committee of 900 Hospital of the Joint Logistics Team (Fuzhou, China) and the Regulations for the Administration of Affairs Concerning Experimental Animals.

SD rats were utilized to evaluate DOX or DOX-BSA/MnO_2_ NPs pharmacokinetics. Female BALB/c mice participated in the building of the 4T1 subcutaneous tumor model for *in vivo* biodistribution. Female BALB/c nude mice were employed for establishing the MCF-7/ADR subcutaneous tumor model for all *in vivo* fluorescence imaging and tumor treatment, besides safety assessment. To create a mouse model of an established tumor, 1 × 10^6^ cells of either the 4T1 or MCF-7/ADR line were suspended in 100 μL of PBS and administered by subcutaneous injection into the left flank region of mice. This equation estimated the tumor volume: V = (W^2^ × L)/2, as V represents tumor volume, W stands for its width, and L stands for its length. V/V_0_ represents the relative volume of the tumor, where V_0_ is the baseline volume prior to medication.

#### 2.2.7 Pharmacokinetic evaluation

SD female rats participated in the *in vivo* pharmacokinetic evaluation of DOX-BSA/MnO_2_ NPs. Therefore, 12 rats were fasted with *ad libitum* water access and split into two groups (each n = 6) randomly, including 1) DOX solution (5 mg/kg); 2) DOX-BSA/MnO_2_ NPs (5 mg/kg equivalent to DOX). Rats were given the DOX solution or the DOX-BSA/MnO_2_ NPs by tail injection. Then, about 500 μL blood was gathered in a 1.5 mL heparinized centrifuge tube at designated time intervals (2, 5, 10, 15, 20, 30, 40, 60, 90, 120, 180, and 300 min). An identical amount of normal saline heated to body temperature was administered intraperitoneally to recover blood volume. Samples were centrifuged at 13,000 rpm for 5 min immediately to recover plasma, which was kept at −80°C for additional processing.

1) Determination of DOX by HPLC

High-pressure liquid chromatography (HPLC) (LC-20A, Shimadzu, Tokyo, Japan) using a fluorescence detector measured DOX concentration in plasma, organs, or tumors. Plasma samples were extracted by precipitation of proteins (acetonitrile: dichloromethane = 1:4) and using daunorubicin (DNR) as an internal standard. The excitation and emission wavelengths used to monitor DOX were 238 and 554 nm, respectively. The mobile phase comprised acetonitrile with 0.1% trifluoroacetic acid (25:75, v/v); online mixing and pumping were performed using a quaternary pump at a 1.0 mL/min flow rate. DOX was separated by a Phenomenex C18 column (250 × 4.6 mm, 5 μm) at 30°C with a 10 μL injection volume. DOX and DNR were eluted in around 3 and 7 min, respectively. The developed HPLC method was verified in the specificity, linearity, precision, accuracy, recovery, limit of detection (LOD), as well as limit of quantitation (LOQ).

A two-compartment model with Phoenix WinNonlin 10.0 program (Pharsight, Mountain View, CA, United States) calculated the pharmacokinetic metrics. The following parameters were estimated: maximum plasma concentration (C_max_), area under the concentration-time curve from baseline to terminal time analyzed (AUC), mean residence time (MRT), clearance rate (Cl), volume of distribution V) and elimination half-life (t_1/2_).

#### 2.2.8 Biodistribution analysis

The 4T1-bearing BALB/c mice took part in studying the tissue distribution. The mice were randomly split into two groups (n = 6), including 1) DOX solution (5 mg/kg); 2) DOX-BSA/MnO_2_ NPs (5 mg/kg equivalent to DOX); administrated with the DOX solution or the DOX-BSA/MnO_2_ NPs *via* tail injection. Six mice from each group were killed at designated time points (1, 8, and 24 h), and the hearts, livers, spleens, kidneys, lungs, and tumors were immediately removed and weighed. About 0.1 g of the tissues were immersed with 1 mL of 0.9% NaCl and then homogenized. DOX was extracted from tissue homogenate by precipitating protein (acetonitrile: dichloromethane = 1:4) and DNR as internal standard and then quantified using HPLC, as Section 2.2.7 illustrates.

#### 2.2.9 Fluorescence animal imaging

The MCF-7/ADR-bearing BALB/c nude mice were distributed randomly into two groups (n = 3). Subsequently, 100 µL of ICG solution or ICG-labeled BSA/MnO_2_ NPs (ICG-BSA/MnO_2_ NPs) were given at 1 mg/kg ICG concentration into the tail vein. Therefore, mice were observed utilizing the IVIS Spectrum CT Imaging System (PerkinElmer, Inc. USA) with fluorescent filter sets (excitation/emission: 740/820 nm) at designated time points (0.5, 1, 2, 4, 6, 8, 12, and 24 h) after injection; then they were sacrificed 24 h after injection. The hearts, livers, spleens, lungs, and kidneys, besides tumors, were gathered; and then imaged utilizing the IVIS Spectrum CT Imaging System under similar circumstances.

#### 2.2.10 *In vivo* therapeutic effectiveness

At tumor volume of about 100 mm^3^, the MCF-7/ADR-bearing BALB/c nude mice were allocated by random into six groups (n = 3), including 1) Saline, 2) DOX, 3) BSA/MnO_2_ NPs, 4) BSA/MnO_2_ NPs + laser irradiation, 5) DOX-BSA/MnO_2_ NPs and 6) DOX-BSA/MnO_2_ NPs + laser irradiation. The DOX and MnO_2_ NPs were injected intravenously (IV) at 1 mg/kg and 0.5 mg/kg doses per mouse, respectively, for each of these formulations. A 635 nm laser with a 1.5 W/cm^2^ power density was also used to irradiate every mouse within the laser-treated group for 10 min and 60 min after injection. Full thermal imaging of the mouse was captured as a thermal infrared camera tracked the temperature change. The formulations above were administered to mice through the IV route on days 1, 4, and 7, and every 2 days for 22 days, the tumor volume and body weight were assessed and documented. Tumors were collected, weighed, and stained using hematoxylin and eosin (HE), TdT-mediated dUTP nick-end labeling (TUNEL), and HIF-1α staining on the 22nd day after all the mice were euthanized. Hearts, livers, spleens, lungs, and kidneys were gathered and stained with HE staining to assess NPs biosafety.

#### 2.2.11 Statistical analyses

The entire analysis was presented as the mean ± SD. Student’s t-test analyzed the results between the two groups. The one-way ANOVA analyzed multiple-group analysis. ^*^ *p* > 0.05 was judged as non-significant. *p < 0.05 was judged significant compared with the corresponding control.

## 3 Results and discussion

### 3.1 Detecting the crystalline state of DOX

**FIGURE 1:**
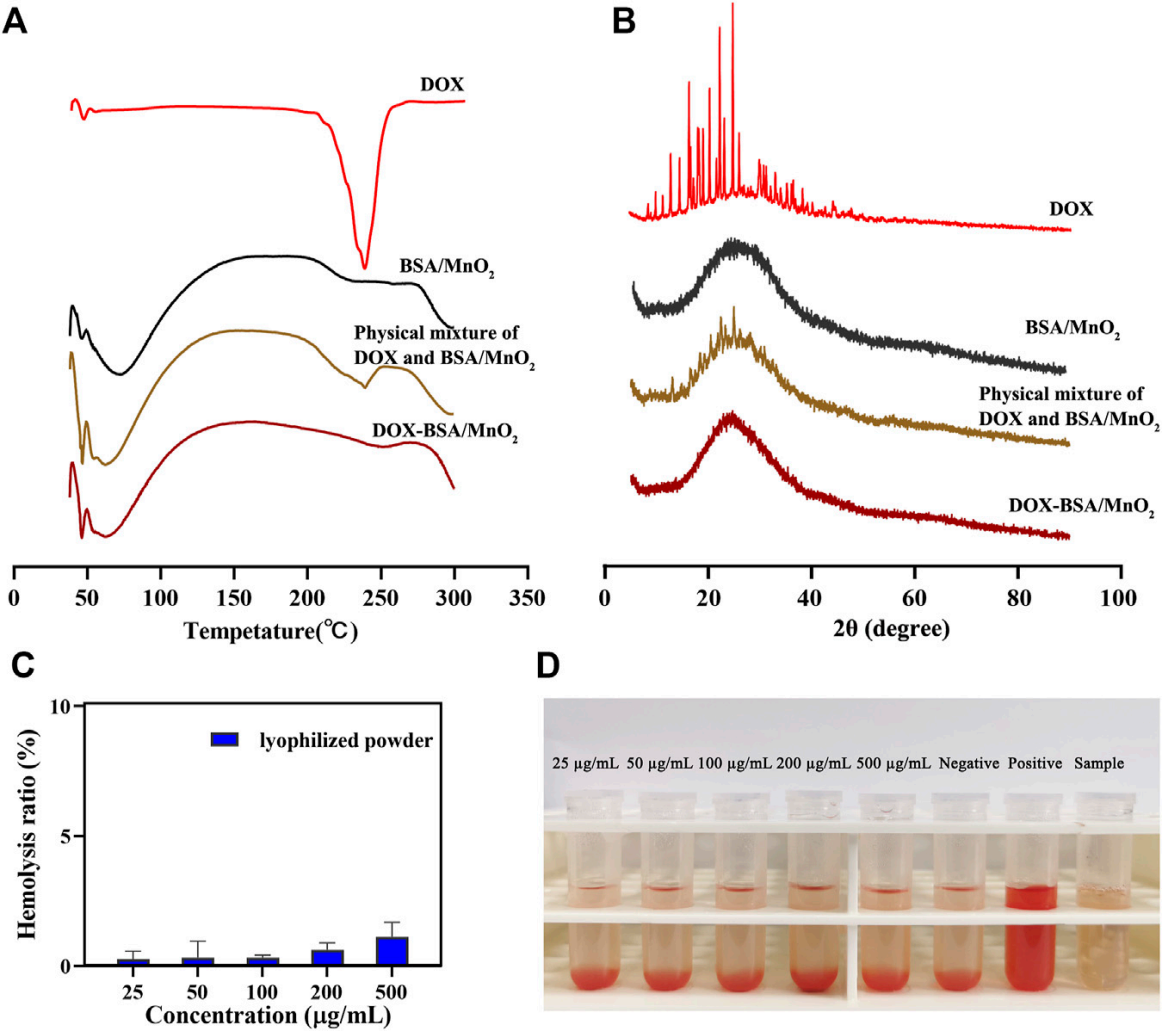
Characterization of NPs. **(A)** Differential scanning calorimetry and; **(B)** X-ray diffraction patterns of DOX, BSA/MnO_2_ NPs, physical mixture of DOX and BSA/MnO_2_ NPs, and DOX-BSA/MnO_2_ NPs; **(C)** hemolysis rate of different concentrations of lyophilized powder of DOX-BSA/MnO_2_ NPs; **(D)** images of hemolytic test results of lyophilized powder of DOX-BSA/MnO_2_ NPs.

The physical state of DOX in DOX-BSA/MnO_2_ NPs can affect multiple medication delivery aspects (Chowdhury et al., 2019). DSC and XRD analyses evaluated the crystalline state of DOX in DOX-BSA/MnO_2_ NPs. Figure 1A depicts the DSC analysis results of DOX, BSA/MnO_2_ NPs, the physical mixture of DOX and BSA/MnO_2_ NPs, together with DOX-BSA/MnO_2_ NPs. Sharp endothermic peaks at 235 C were presented in the physical mixture of DOX and BSA/MnO_2_ NPs because of the melting points of DOX crystals. However, as BSA/MnO_2_ NPs, this peak was unobserved in DOX-BSA/MnO_2_ NPs.

For XRD analysis (Figure 1B), sharp characteristic peaks of DOX were detected at 2θ measures in the range of 16°**–**27° due to the high crystalline characteristics of DOX (Eskitoros-Togay et al., 2019; Permana et al., 2020). However, the sharp characteristic peaks of DOX were unobserved in DOX-BSA/MnO_2_ NPs, consistent with DSC results. DOX peak absence in DSC and XRD analysis suggested that DOX presented as amorphous in DOX-BSA/MnO_2_ NPs, which may be because of DOX complete encapsulation in BSA/MnO_2_ NPs.

### 3.2 Hemolysis analysis

Hemolysis is a proven technique to evaluate the biocompatibility property of NPs (Sankar and Ravikumar, 2014). Therefore, the hemolysis behavior was first investigated to determine the possible toxicity of DOX-BSA/MnO_2_ NPs throughout the vascular circulation *in vivo* investigation. Less than 2% hemolysis occurred at the highest DOX-BSA/MnO_2_ NPs concentration (500 μg/mL), demonstrating the good hemocompatibility of DOX-BSA/MnO_2_ NPs (Figures 1C, D).

### 3.3 Cytotoxicity analysis

**FIGURE 2:**
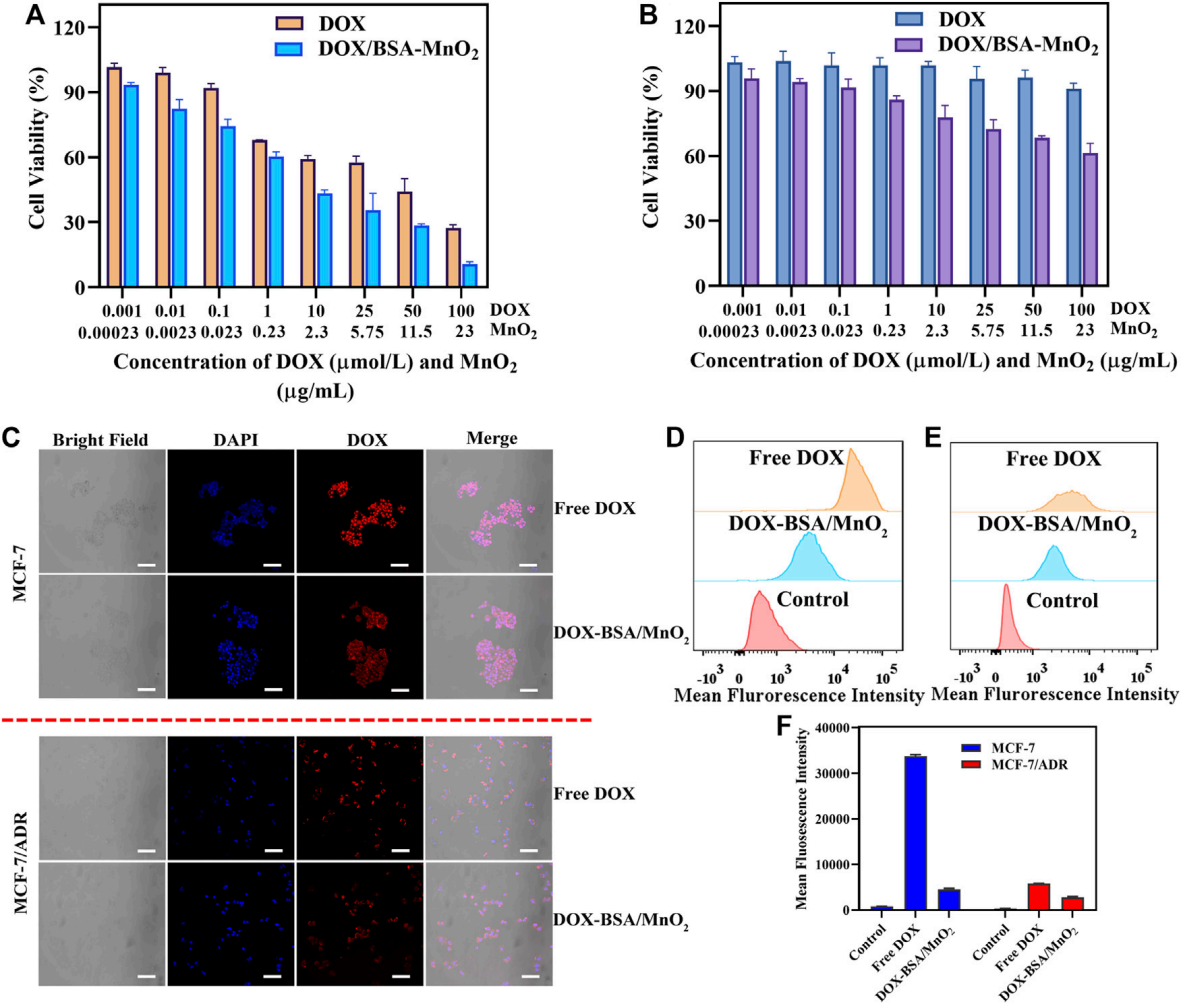
*In vitro* cellular experiments. The cell viability of **(A)** MCF-7 cells as well as; **(B)** MCF-7/ADR cells subjected to DOX along with DOX-BSA/MnO_2_ NPs of several doses; n = 5; **(C)** cellular uptake of free DOX besides DOX-BSA/MnO_2_ NPs by MCF-7 together with MCF-7/ADR cells after incubation for 6 h; scale bar = 100 μm; **(B)** overlay of fluorescence intensity data obtained through flow cytometer of **(D)** MCF-7 cells as well as; **(E)** MCF-7/ADR cells incubated with DOX along with DOX-BSA/MnO_2_ NPs for 6 h; **(C)** mean fluorescence intensity of MCF-7 cells besides MCF-7/ADR cell lines incubated with DOX-BSA/MnO_2_ NPs for 6 h; n = 3.

MTT assay determined the *in vitro* cytotoxicity. Figures 2A, B illustrate that both DOX and DOX-BSA/MnO_2_ NPs possess a dose-dependent inhibitory impact against MCF-7 and MCF-7/ADR cells. Moreover, free DOX presented significant cytotoxicity to MCF-7 cells at 0.001–100 μM (IC50 = 26.0 μM) concentrations, unlike MCF-7/ADR cells (IC50 = 160.1 μM) as cytotoxicity was much lower, assuring the MCF-7/ADR cells to be DOX resistant. On contrary to free DOX treatment, DOX-BSA/MnO_2_ NPs demonstrated reinforced cytotoxicity under the same conditions with an IC50 of 11.0 μM in MCF-7 cells and an IC50 of 54.7 μM in MCF-7/ADR cells. These outcomes stated that BSA/MnO_2_ NPs were promising carriers to enhance the DOX chemotherapy effect and could effectively reverse DOX resistance *in vitro.*

### 3.4 *In vitro* cellular uptake investigation

The efficacy of anti-cancer drugs relies on their intracellular accumulation of drugs, particularly in drug resistance cancer cells (Li J. et al., 2022). Therefore, confocal fluorescence microscopy (CLSM) was used to visualize the drug uptake in MCF-7 and MCF-7/ADR cells following 6 h of incubation, while the flow cytometer quantified the mean fluorescence intensity.

Figure 2C illustrates that the red fluorescence was much lower in DOX-BSA/MnO_2_ NP-treated cells contrasted with the free-DOX-treated cells, indicating the cell internalization of DOX-BSA/MnO_2_ NPs was significantly slower than free DOX, which may be because of the difference between DOX-BSA/MnO_2_ NPs endocytosis along with DOX passive diffusion mechanism and different DOX release rate of DOX together DOX-BSA/MnO_2_ NPs (Wallat et al., 2018; Zhang et al., 2021). DOX acts mainly by entering the nucleus and causing DNA damage (Yang et al., 2022). The DOX fluorescence was mainly accumulated in MCF-7 cell nuclei following incubation with free DOX along with DOX-BSA/MnO_2_ NPs. However, DOX fluorescence was mainly detected in MCF-7/ADR cells cytoplasm following the free DOX incubation, which may be caused by drug efflux, the key reason for drug resistance (Li et al., 2019). Notably, DOX-BSA/MnO_2_ NPs have mainly localized in the MCF-7/ADR cell nuclei, increasing the potential to reverse resistance by reducing drug efflux.

According to the CLSM findings, the cellular fluorescence intensities of MCF-7 and MCF-7/ADR cells subjected to free DOX were approximately seven and two times greater, respectively, compared to those following DOX-BSA/MnO_2_ NPs therapy (Figures 2D–F). These findings showed the drug uptake effectiveness was entirely inequivalent to the drug actions, implying more factors contribute to NPs enhanced cytotoxicities, such as endocytosis, distribution, increased apoptosis, or modified molecular mechanisms of action (Zeng et al., 2014).

### 3.5 Pharmacokinetic and biodistribution evaluation

#### 3.5.1 Validating HPLC method for quantitative analysis of DOX

Pharmacokinetic and biodistribution studies were performed to obtain more insight into the difference between the DOX-BSA/MnO_2_ NPs and the DOX solution. The HPLC technique was employed to detect the amount of DOX in plasma, organs, and tumors. The method was verified regarding specificity, linearity, precision, accuracy, recovery, LOD, and LOQ.

Supplementary Figure S1–S7 illustrate no obvious interference in the representative chromatogram of all the analytes, indicating the specificity of method for DOX. For the linearity study, DOX concentrations (50–2000 ng/mL) had good linearity and correlation coefficients (*r* > 0.99) (Supplementary Table S1). Moreover, the LOD and LOQ were observed as 20 ng/mL and 50 ng/mL in plasma, organs, or tumors, respectively. The recovery of DOX in plasma, organs, or tumors was between 82.31% and 97.41%, with RSD between 1.10% and 4.69% in the concentration of 50 ng/mL, 500 ng/mL, and 2000 ng/mL (Supplementary Table S2), meeting the requirements of the analysis method of biological samples. The intra- and inter-day precisions, along with accuracies results of DOX in plasma, organs, or tumors at different concentrations (Supplementary Tables S3–S5). The RSD of precisions were all <5%, and accuracies were all >90%. The findings suggested that the technique was accurate and precise enough. The evolved HPLC technique was applied to measure DOX concentration in plasma, organs, or tumors.

#### 3.5.2 Pharmacokinetic evaluation

**FIGURE 3:**
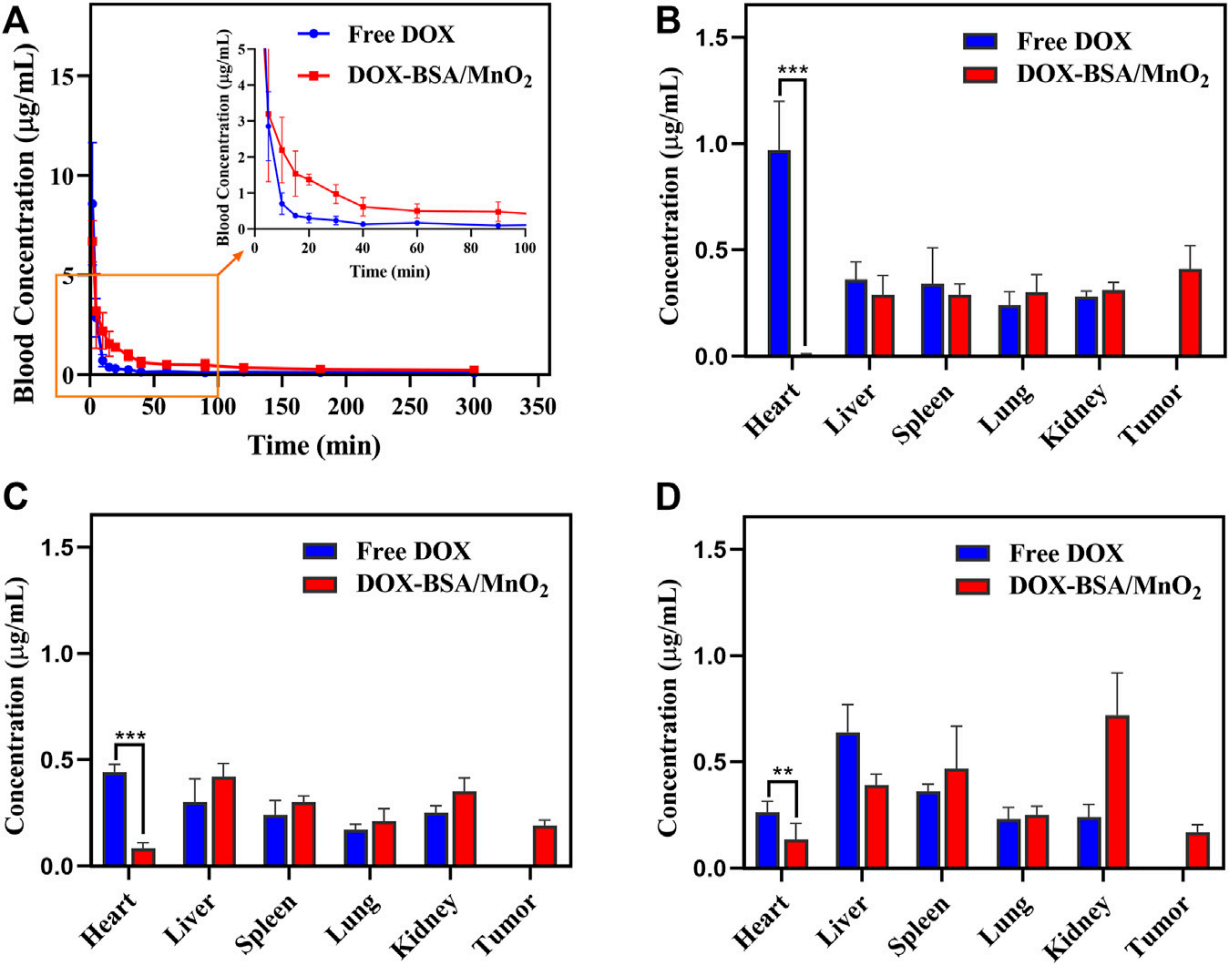
**(A)** Plasma concentration-time curve of DOX solution along with DOX-BSA/MnO_2_ NPs in rats after single IV injection; n = 6; concentration in different organs and tumors **(B)** at 1 h; **(C)** 8 h and; **(D)** 24 h after IV injection of free DOX solution together with DOX-BSA/MnO_2_ NPs in mice; n = 6.

Figure 3A presents the plasma concentration-time curves of DOX, as well as DOX-BSA/MnO_2_ NPs. Table 1 summarizes the two-compartment model pharmacokinetic parameters calculated using the WinNonlin program. Free DOX had an *in vivo* elimination half-life (t_1/2_) on the minute order, with a systemic clearance that is so quick that 15 min following IV injection, DOX concentration in the blood was <5% of the initial one. On the contrary, the DOX-BSA/MnO_2_ NPs had a much longer t_1/2_ of 0.45 ± 0.093 h, which was 3.88 times higher than that of DOX (0.093 ± 0.028 h) (*p* < 0.05), indicating that the DOX-BSA/MnO_2_ NPs could prolong the circulation time of DOX *in vivo*. The AUC of DOX-BSA/MnO_2_ NPs was (3539.83 ± 422.61 h‧ng/mL), which was increased 2.02-fold more than that of DOX (1749.61 ± 336.11 h‧ng/mL) (*p* < 0.05). Furthermore, the plasma kinetics of DOX-BSA/MnO_2_ NPs showed significantly lower maximum concentration in plasma (C_max_) than that of DOX (58.46% reduction), demonstrating that DOX-BSA/MnO_2_ NPs avoided DOX side effects caused by excessive blood concentration. Furthermore, DOX-BSA/MnO_2_ NPs exhibited decreased Cl and increased V and MRT compared to free DOX, implying longer NPs retention in blood circulation. The longer systemic circulation time of DOX-BSA/MnO_2_ NPs revealed the sustained DOX release from NPs, which is considered crucial for enhancing treatment effectiveness (Parhizkar et al., 2022). Therefore, DOX-BSA/MnO_2_ NPs could significantly alter DOX pharmacokinetic properties, which was beneficial for its *in vivo* application.

**TABLE 1:** Pharmacokinetic metrics of DOX solution besides DOX-BSA/MnO_2_ NPs after a single IV in rats, n = 6.

Pharmacokinetics parameters	DOX	DOX-BSA/MnO_2_ NPs
t_1/2_ (h)	0.093 ± 0.028	0.45 ± 0.093
AUC (h*ng/mL)	1749.61 ± 336.11	3539.83 ± 422.61
C_max_ (ng/mL)	13,024.79 ± 3920.26	5409.99 ± 890.13
Cl (mL/h/kg)	2.90 ± 0.55	1.40 ± 0.17
V (mL/kg)	0.38 ± 0.12	0.92 ± 0.15
MRT (h)	2.13 ± 1.15	3.12 ± 1.99

Note: Results reported as mean ± SD (n = 6). t_1/2_: elimination half-life; AUC: area under the concentration-time curve from time 0 to the last time analyzed; C_max_: maximum plasma concentration; Cl: clearance rate; V: volume of distribution; MRT: mean residence time.

#### 3.5.3 Biodistribution analysis

To investigate the biodistribution of DOX-BSA/MnO_2_ NPs, hearts, livers, spleens, lungs, kidneys, and tumor tissues were gathered at 1, 8, and 24 h post-injection. DOX equivalent levels were determined for each period. As a control, the same dose of free DOX was administered. The DOX concentration was detected at the tumor location following 24 h of injecting DOX-BSA/MnO_2_ NPs (Figures 3B–D). In contrast, it could not be measured after injection of DOX solution, revealing the enhanced tumor targeting capability of DOX-BSA/MnO_2_ NPs compared with free DOX, which was expected to provide higher antitumor efficacy. This prolonged tumor retention behavior of DOX-BSA/MnO_2_ NPs was probably caused by the EPR impact due to the drug carriers in a narrow size range from about 10 to 100 nm (Petros and DeSimone, 2010) and through active targeting mediated by gp60-cellar protein-SPARC (Noorani et al., 2015; Tan et al., 2021).

The effectiveness of DOX is usually limited because of its cardiac toxicity (Wei et al., 2020). Fortunately, a decreased DOX accumulation was detected in the hearts for DOX-BSA/MnO_2_ NPs compared with the DOX solution, revealing the DOX-BSA/MnO_2_ NPs’ ability to reduce cardiac toxicity and adverse effect of DOX. A great quantity of DOX was detected in the liver after injecting DOX solution IV, suggesting that DOX was mainly metabolized in the liver. However, more DOX was measured in the kidney following injecting DOX-BSA/MnO_2_ NPs IV, revealing that DOX-BSA/MnO_2_ NPs could alter DOX metabolized way. The tiny size of the BSA-templated NPs makes passive aggregation at the tumor location and metabolism through the kidney easier (Xu et al., 2018).

### 3.6 Fluorescence imaging of animals

**FIGURE 4:**
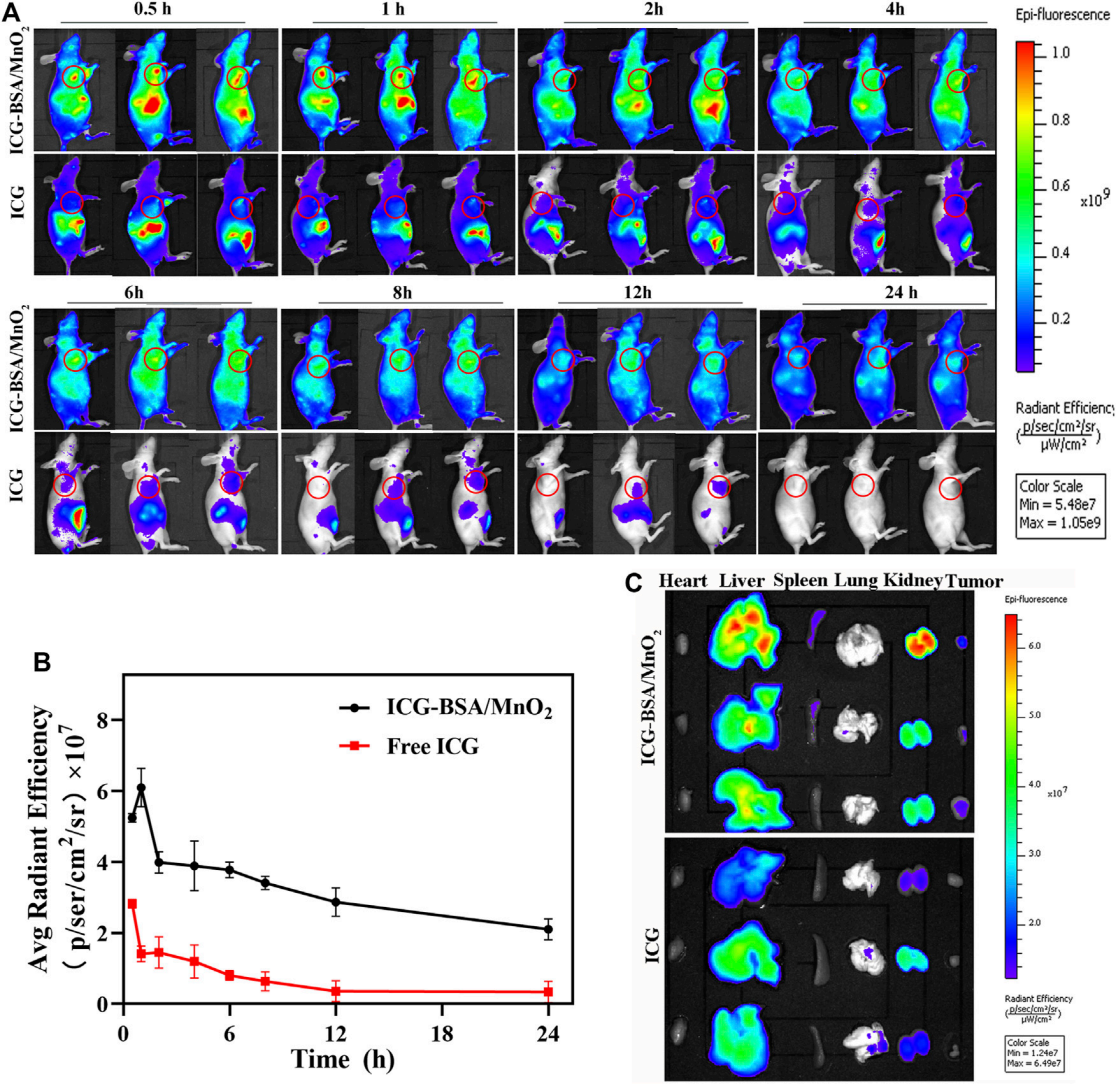
*In vivo* as well as *ex vivo* biodistribution of free ICG and ICG-BSA/MnO_2_ NPs following IV injection. **(A)** *In vivo* entire-animal imaging of ICG fluorescence at several time points (0.5, 1, 2, 4, 6, 8, 12, and 24 h) following IV administration of free ICG and ICG-BSA/MnO_2_ NPs (the tumor zone is circled in red); **(B)** measuring the fluorescence intensity of tumors in MCF-7/ADR-bearing BALB/c nude mice at developed time points (0.5, 1, 2, 4, 6, 8, 12, as well as 24 h) after IV injection of free ICG along with ICG-BSA/MnO_2_ NPs; **(C)** *Ex-vivo* imaging of main organs and tumors distribution of ICG following IV administration of free ICG and ICG-BSA/MnO_2_ NPs for 24 h.

Improved accumulation at the tumor site is critical to the ultimate treatment outcome. Therefore*, in vivo* fluorescence imaging determined the optimal laser irradiation time and investigated NPs’ targeting capability. BSA/MnO_2_ NPs were labeled with ICG (1 mg/kg) (ICG-BSA/MnO_2_ NPs), and the synthesis process was mentioned before (Chen et al., 2022). Additionally, an identical dose of free ICG was used as a control. The fluorescence intensity of ICG-BSA/MnO_2_ NPs at the tumor sites in MCF-7/ADR-bearing nude mice increased and then decreased, and the peak fluorescence intensity occurred 1 h after injection (Figures 4A, B). Moreover, the fluorescence signal persists for more than 24 h after injection. In contrast, free ICG fluorescence intensity reduces rapidly with time following the free ICG injection, revealing that free ICG would be quickly cleared from the bloodstream.

Moreover, the major organs and tumors were gathered, and their fluorescence intensity was examined after 24 h of administration. The fluorescence intensity of the ICG-BSA/MnO_2_ NPs group was elevated compared to the free ICG group at the tumor sites (Figure 4C). The results demonstrated that ICG-BSA/MnO_2_ NPs had a long circulation time and good targeting capacity, improving the *in vivo* therapeutic effect. Moreover, the kidneys in the ICG-BSA/MnO_2_ NPs group and the livers in the free ICG group showed stronger fluorescence signals, respectively, indicating that the kidney metabolized NPs mainly while the liver metabolized the free ICG. These findings were consistent with those of biodistribution in Section ′3.5.3′, and the optimal PTT treatment time was 1 h after drug administration.

### 3.7 *In vivo* therapeutic effectiveness

**FIGURE 5:**
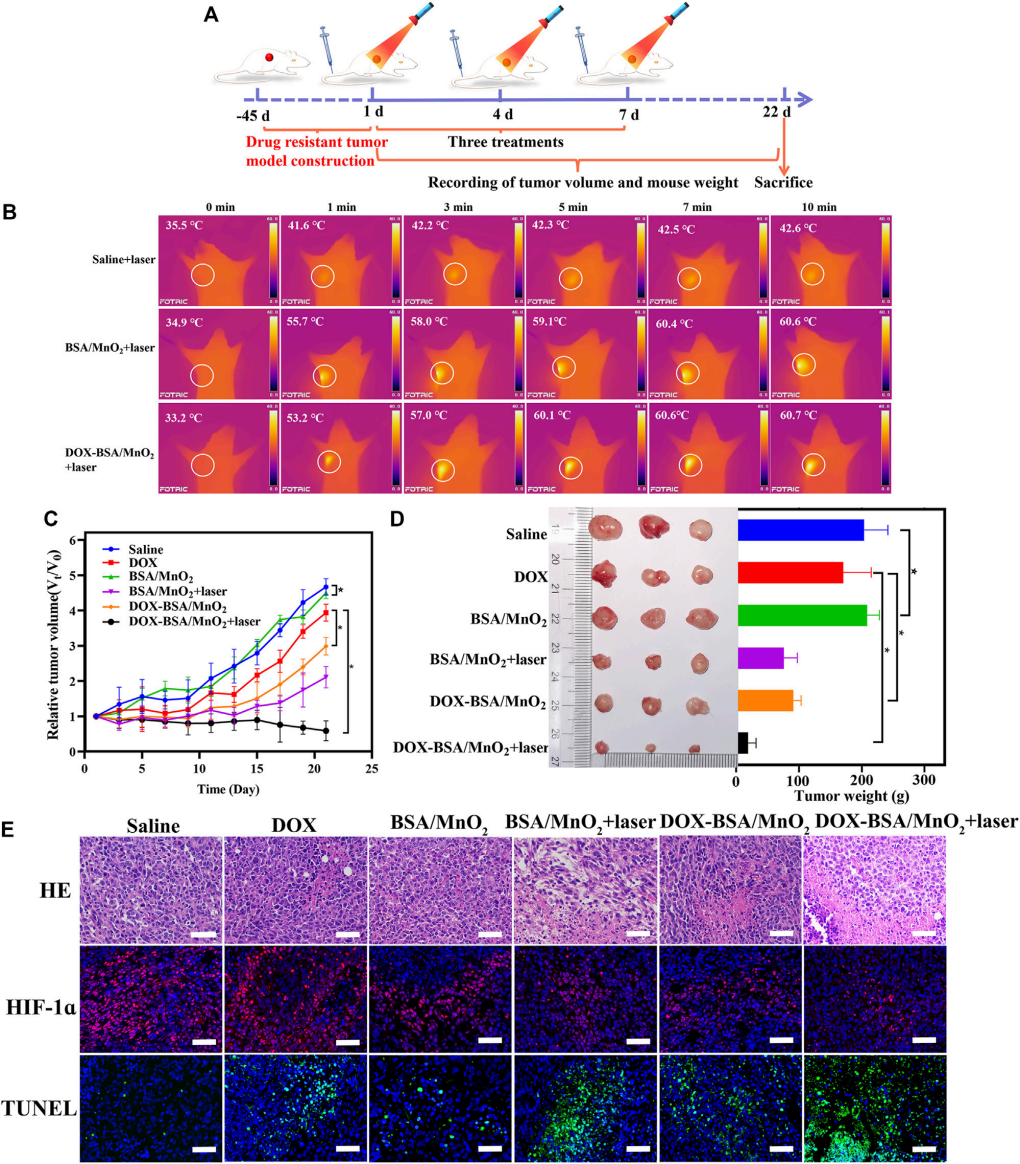
*In vivo* antitumor effectiveness of NPs on MCF-7/ADR bearing BALB/c nude mice. **(A)** Diagrammatic representation of the experimental animal design; **(B)** thermal *in vivo* photos and tumor temperature of mice under 635 nm laser irradiation (1.5 W/cm^2^, 10 min); **(C)** relative tumor volume change curves of mice following various therapies; n = 3; **(D)** visual observations of tumor sizes and weights at the end time point; n = 3; **(E)** representative images of HE; TUNEL; and HIF-1α staining of the tumor following various therapies on the 22nd day; nuclei in blue (DAPI staining); apoptotic cells in green (TUNEL staining); and hypoxic cells in red (HIF-1α staining); scale bar = 50 μm.

BALB/c nude mice bearing MCF-7/ADR cell line were employed to explore whether the DOX-BSA/MnO_2_ NP-mediated hypoxia ameliorated and photothermal effect would reverse the DOX resistance. At approximately 100 mm^3^ tumor volumes, the mice were allocated by random to six groups (n = 3), medicated thrice with saline, DOX, BSA/MnO_2_ NPs, BSA/MnO_2_ NPs + laser irradiation, DOX-BSA/MnO_2_ NPs, or DOX-BSA/MnO_2_ NPs + laser irradiation on days 1, 4, and 7 (Figure 5A). Both MnO_2_, as well as DOX doses, were 0.5 mg/kg and 1 mg/kg, respectively, with 635 nm laser (1.5 W/cm^2^, 10 min) tumors irradiation at 1 h after injection in the laser treatment group.

The temperature change was obtained *via* infrared thermal imaging to visually investigate the temperature fluctuation of MCF-7/ADR-bearing nude mice of DOX-BSA/MnO_2_ NPs *in vivo.* The surface temperature of tumor site in mice administrated saline was non-significantly increased and only from 35.5°C to 42.6°C under 635 nm laser irradiation for 10 min (Figure 5B). In contrast, the irradiated tumor tissues of BSA/MnO_2_ NP- together with DOX-BSA/MnO_2_ NP-treated mice showed a significant temperature elevation. The surface temperature of tumor increased from 34.9°C to 60.6°C and 33.2°C–60.7°C, respectively, which was high enough to kill malignant cells (Dun et al., 2022; Tian et al., 2022), demonstrating DOX-BSA/MnO_2_ NPs exhibited good targeting to drug resistance breast cancer cells, allowing more NPs to aggregate at the tumor location and exert better PTT effects.

Subsequently, we evaluated the capability of DOX-BSA/MnO_2_ NPs to suppress DOX-resistant tumor proliferation *in vivo*. Compared with the saline-treated group, the weak antitumor ability of BSA/MnO_2_ NPs was observed since MnO_2_ produces free radicals (Figure 5C), which destroy cancer (Veroniaina et al., 2021). With BSA/MnO_2_ NPs + laser irradiation, the tumor volume exhibited a significant reduction, indicating that hyperthermia could effectively cause cell ablation. Tumor growth in the DOX treatment group was marginally limited due to the inferior tumor-targeted ability of DOX (Huang et al., 2019). Interestingly, at the same concentration of DOX, DOX-BSA/MnO_2_ NPs showed a significant reduction in tumor development than free DOX. DOX was less efficient in hypoxic than normoxic environments (Zeng et al., 2021). Therefore, the enhancement of antitumor action of DOX-BSA/MnO_2_ NPs as a result of the MnO_2_ for reliving tumor hypoxia. DOX-BSA/MnO_2_ NPs plus laser irradiation medicated group had the greatest therapeutic activity with minimal tumor volume after 22 days of treatment due to the excellent PTT effect and O_2_-generating ability of BSA/MnO_2_ NPs. The tumor mass was removed after the therapy was completed following a similar pattern (Figure 5D).

Additionally, the antitumor efficacy of DOX-BSA/MnO_2_ NPs was assessed by HE staining and TUNEL assay (Figure 5E). Similar to tumor growth inhibition, HE staining results showed that most tumor cells exhibited obvious nuclear damage after DOX-BSA/MnO_2_ NPs + laser irradiation treatment. TUNEL staining also presented the highest level of tumor cell apoptosis of DOX-BSA/MnO_2_ NPs + laser irradiation group, as demonstrated by the brightest green fluorescent signals at tumor sites.

HIF-1α is crucial in drug resistance, which would be activated in a hypoxic environment and overexpressed within distinct common solid malignancies, such as breast, colon, and gastric (Wigerup et al., 2016; Shukla et al., 2017). Accordingly, DOX-BSA/MnO_2_ NPs’ capability to control hypoxic conditions in tumor tissue was investigated *via* HIF-1α immunofluorescence histochemistry. HIF-1α was more downregulated in the O_2_-generating groups than the saline-treated mice, including the group of BSA/MnO_2_ NPs, BSA/MnO_2_ NPs + laser irradiation, DOX-BSA/MnO_2_ NPs, and DOX-BSA/MnO_2_ NPs + laser irradiation, as demonstrated by the significantly reduced hypoxia signals indicated by HIF-1α antibody staining (Figure 5E). Additionally, further downregulation of HIF-1α was observed after NPs plus laser irradiation, proving that photothermal heating could improve vascular perfusion, cell membrane permeability, and tumor hypoxia (Tang et al., 2019). The results demonstrated that BSA/MnO_2_ NPs or DOX-BSA/MnO_2_ NPs were administered to the hypoxic tumor region, followed by a reaction with H_2_O_2_ and H^+^ to release O_2_ effectively. Hypoxia alleviation led to HIF-1α inhibition.

**FIGURE 6:**
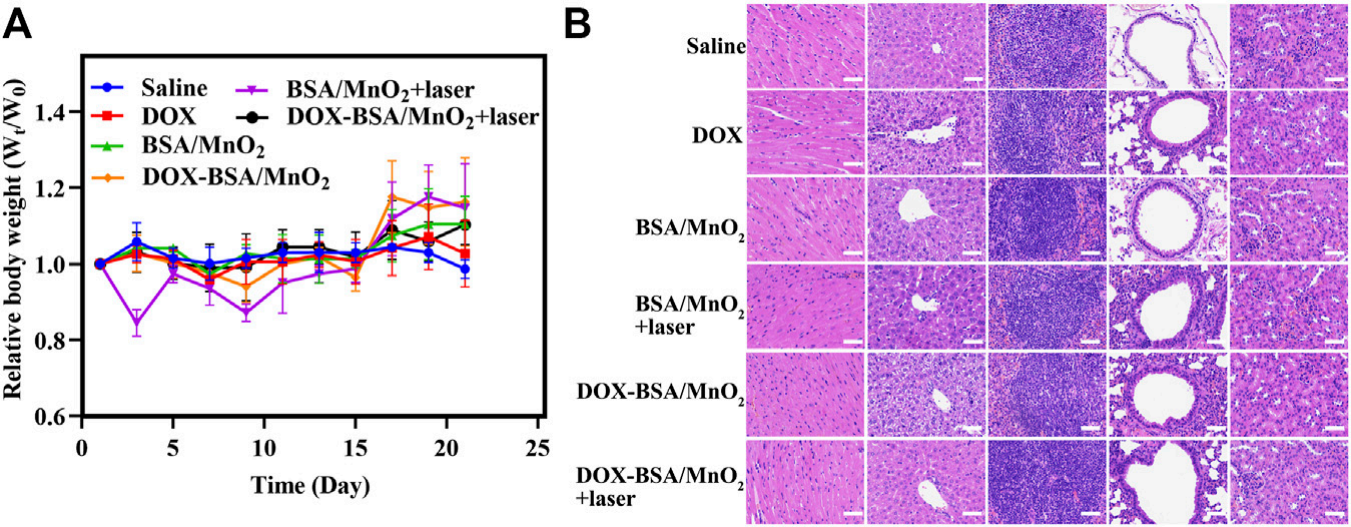
Biosafety evaluation. **(A)** The relative body weight alteration of MCF-7/ADR tumor-bearing nude mice following medications with various formulations; n = 3; **(B)** H&E staining photos of hearts; livers; spleens; lungs; and kidneys of MCF-7/ADR tumor-bearing nude mice after therapies; scale bar = 50 μm.

The body weight was assessed during treatment to test DOX-BSA/MnO_2_ NPs biosafety. Until the end of the observation, the body weight showed a non-significant difference (Figure 6A). Moreover, the HE staining of the main organs in all treatment groups revealed inapparent inflammatory damage and tissue damage, indicating the good biocompatibility of NPs (Figure 6B). Finally, these *in vivo* findings indicated that DOX-BSA/MnO_2_ NPs could reverse DOX resistance showing better anticancer activity as well as biocompatibility.

## 4 Conclusion

The hypoxia-ameliorated photothermal MnO_2_ platform we developed (DOX-BSA/MnO_2_ NPs) successfully reversed the DOX resistance in breast cancer. MnO_2_ in DOX-BSA/MnO_2_ reacted with H_2_O_2_ together with H^+^ in TME to generate O_2_, leading to tumor-hypoxia overcome. Moreover, hyperthermia induced by BSA/MnO_2_ NPs relieved hypoxia, reversing DOX resistance by HIF-1α expression downregulation and improving the antitumor efficiency. Besides, DOX-BSA/MnO_2_ NPs demonstrated excellent biocompatibility and hemocompatibility, which were suitable for their *in vivo* application. Meanwhile, DOX-BSA/MnO_2_ NPs could prolong circulation time *in vivo*, enhance tumor site accumulation, and reduce the cardiotoxicity of DOX, resulting in enhanced therapeutic efficiency. Overall, the as-prepared DOX-BSA/MnO_2_ nanoplatform was a promising therapeutic agent for reversing DOX reversal for better clinical outcomes.

## Data Availability

The original contributions presented in the study are included in the article/Supplementary Material, further inquiries can be directed to the corresponding authors.
