# Supplementary material for: Hypoxia-ameliorated photothermal manganese dioxide nanoplatform for reversing doxorubicin resistance
Source: Front Pharmacol. 2023 Feb 24;14:1133011. doi: 10.3389/fphar.2023.1133011 (PMC9998484; doi:10.3389/fphar.2023.1133011)
Supplement: Supplementary file 1 [file DataSheet1.docx]

Supplementary Material

Hypoxia-Ameliorated Photothermal Manganese Dioxide Nanoplatform for Reversing Doxorubicin Resistance

Zhenzhen Chen^a,1^, Zhihong Liu^b,1^, Qian Zhang^c^, Sheng Huang^a^, Zaizhong Zhang^a^, Xianquan Feng^c^, Lingjun Zeng^b^, Ding Lin^d^, Lie Wang^a,*^, Hongtao Song^b,*^

a Department of General Surgery, 900TH Hospital of Joint Logistics Support Force, Fuzhou 350025, PR China

b Department of Pharmacy, 900TH Hospital of Joint Logistics Support Force, Fuzhou 350025, PR China

c College of Pharmacy, Fujian Medical University, Fuzhou 350108, PR China

d Department of Pharmacy, Jiaxing Maternal and Child Health Care Hospital, Affiliated Hospital of Jiaxing University, Jiaxing 314000, P.R. China

1: These authors contributed equally to this paper

*** Correspondence:**Lie Wang: Fax: +86-0591-2285-9770; Mobile phone: +86-139-0501-5332

E-mail: fzptwk@126.com

Hongtao Song: Fax: +86-0591-2285-9459; Mobile phone: +86-138-5015-2727

E-mail: sohoto@vip.163.com

# Supplementary Figures and Tables

## Supplementary Figures


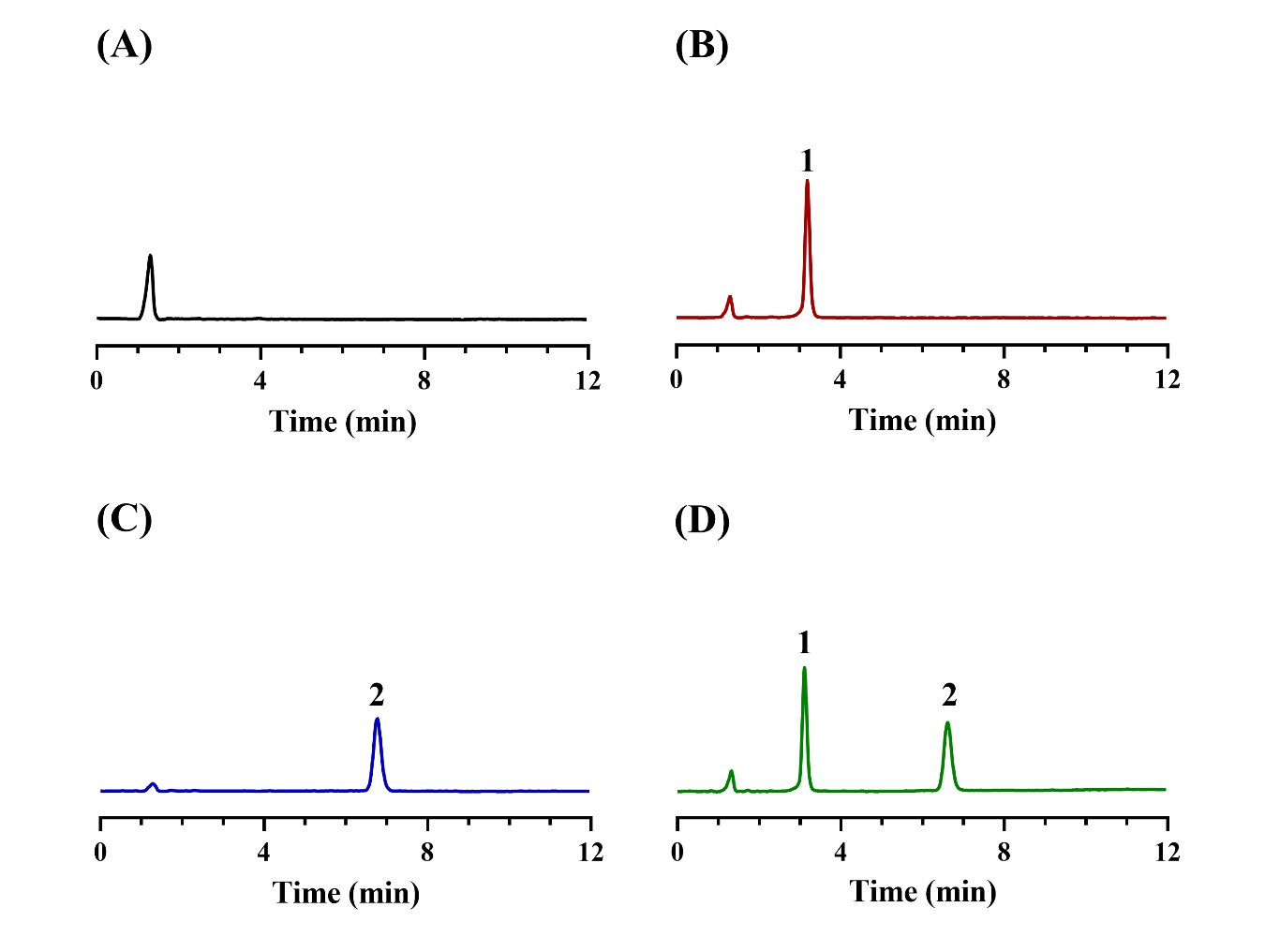


**Figure S1** Chromatograms of (A) blank plasma, (B) blank plasma with DOX solution, (C) blank plasma with DNR solution, and (D) blank plasma with DOX and DNR solution (Note: where 1 refers to blank plasma, 2 refers to DOX solution, 3 refers to DNR solution)


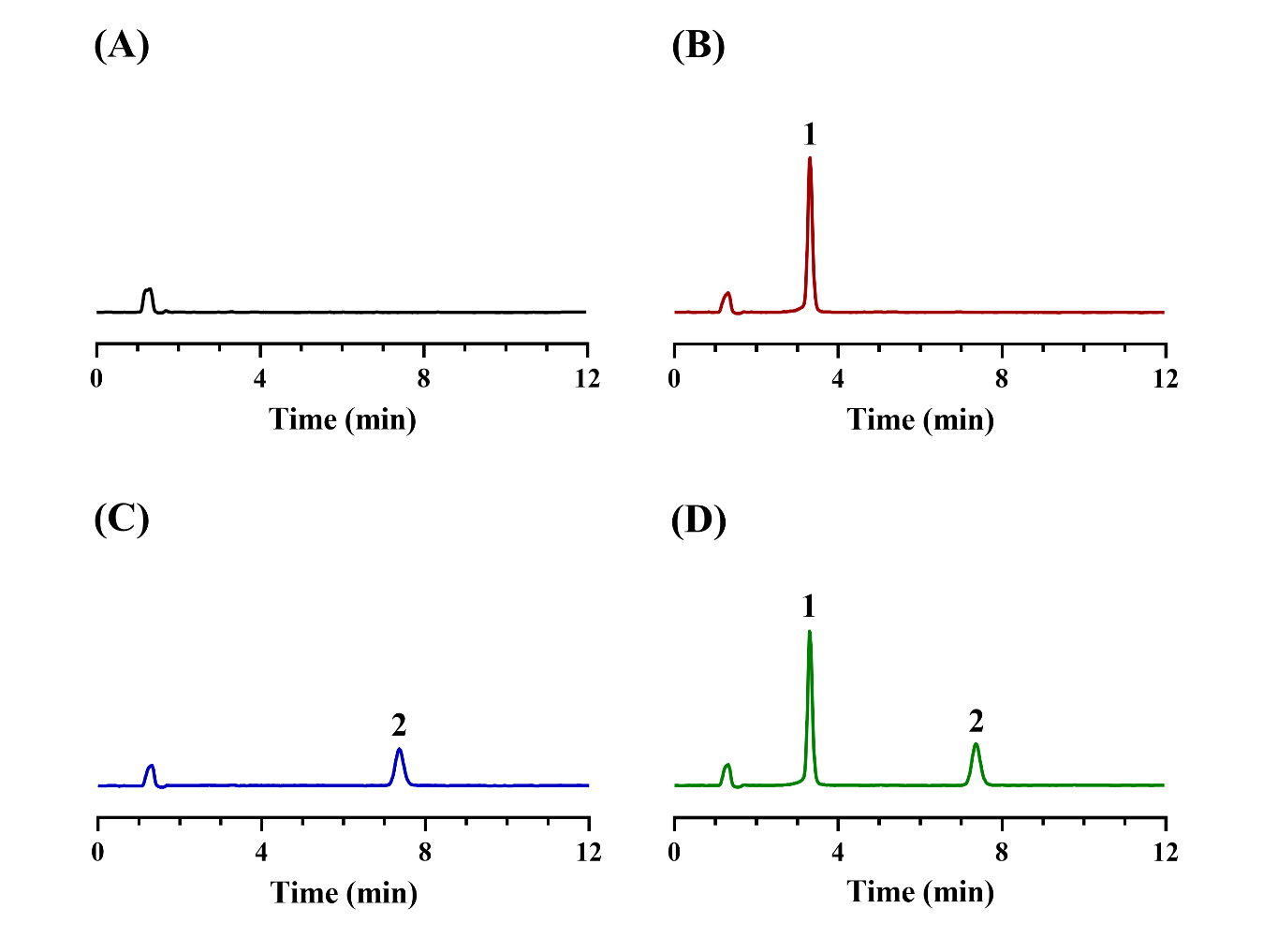


**Figure S2** Chromatograms of (A) blank mouse heart, (B) blank mouse heart with DOX solution, (C) blank mouse heart with DNR solution, and (D) blank mouse heart with DOX and DNR solution (Note: where 1 refers to blank mouse heart, 2 refers to DOX solution, 3 refers to DNR solution)


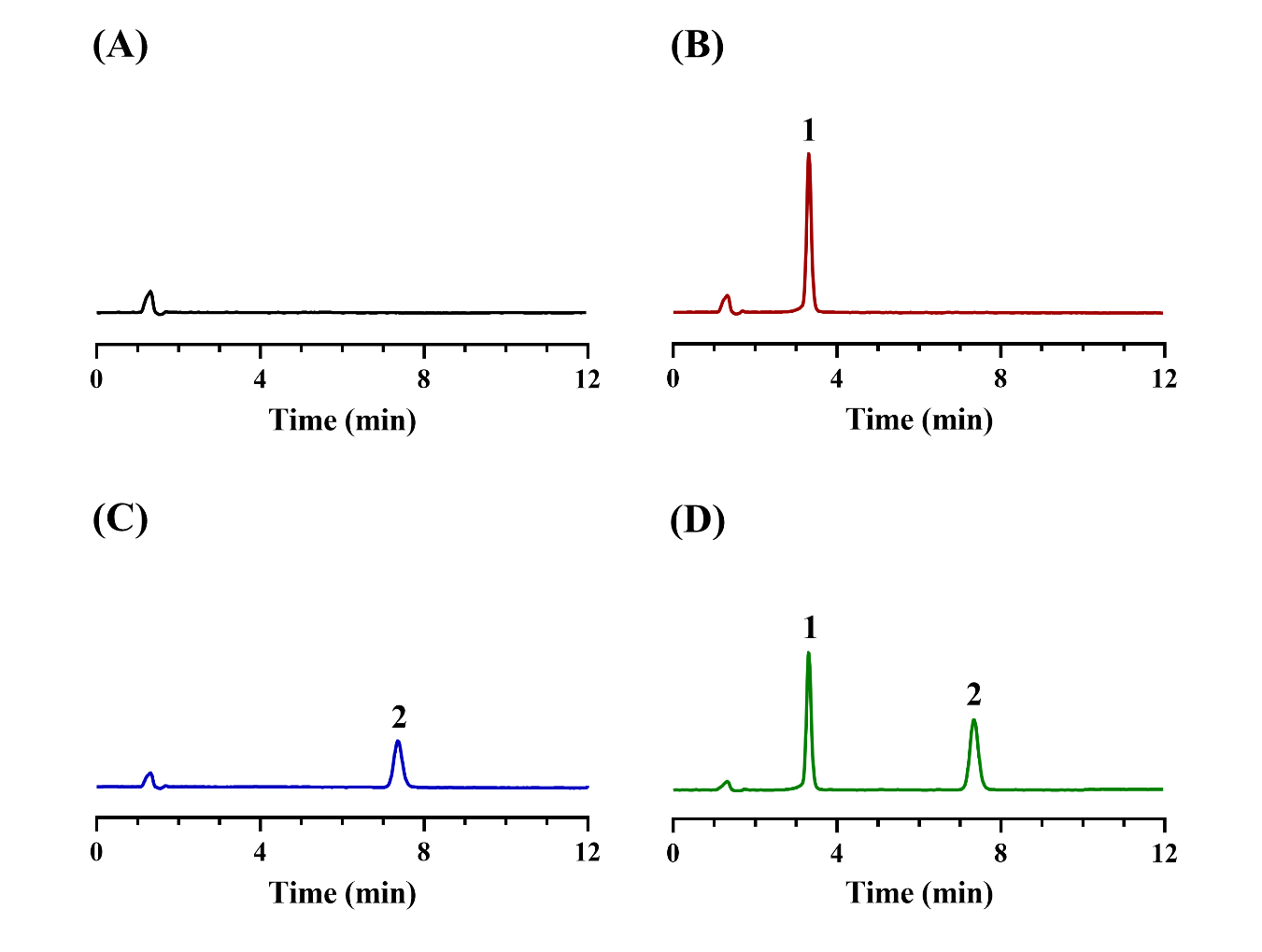


**Figure S3** Chromatograms of (A) blank mouse liver, (B) blank mouse liver with DOX solution, (C) blank mouse liver with DNR solution, and (D) blank mouse liver with DOX and DNR solution (Note: where 1 refers to blank mouse liver, 2 refers to DOX solution, 3 refers to DNR solution)


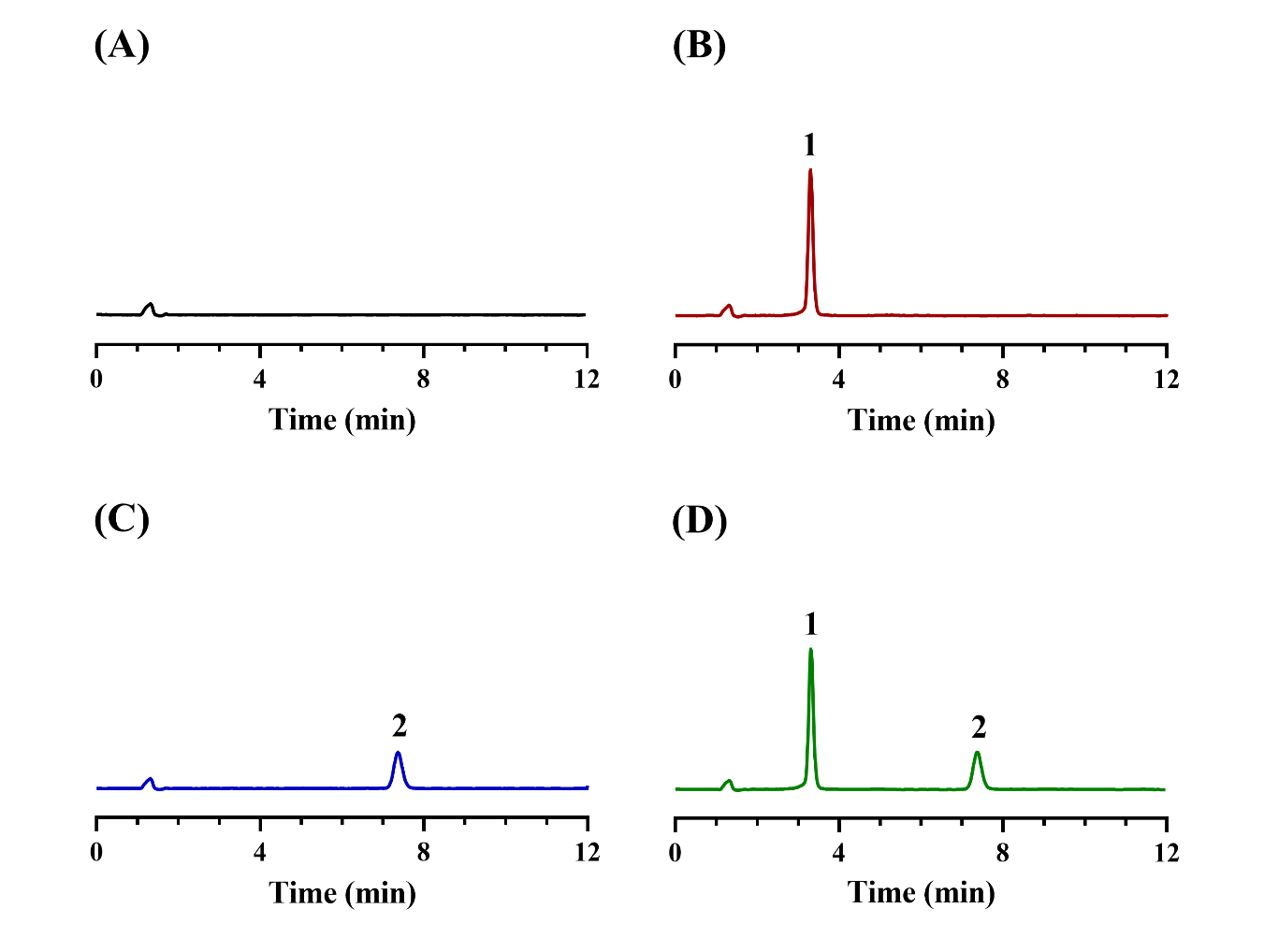


**Figure S4** Chromatograms of (A) blank mouse spleen, (B) blank mouse spleen with DOX solution, (C) blank mouse spleen with DNR solution, and (D) blank mouse spleen with DOX and DNR solution (Note: where 1 refers to blank mouse spleen, 2 refers to DOX solution, 3 refers to DNR solution)


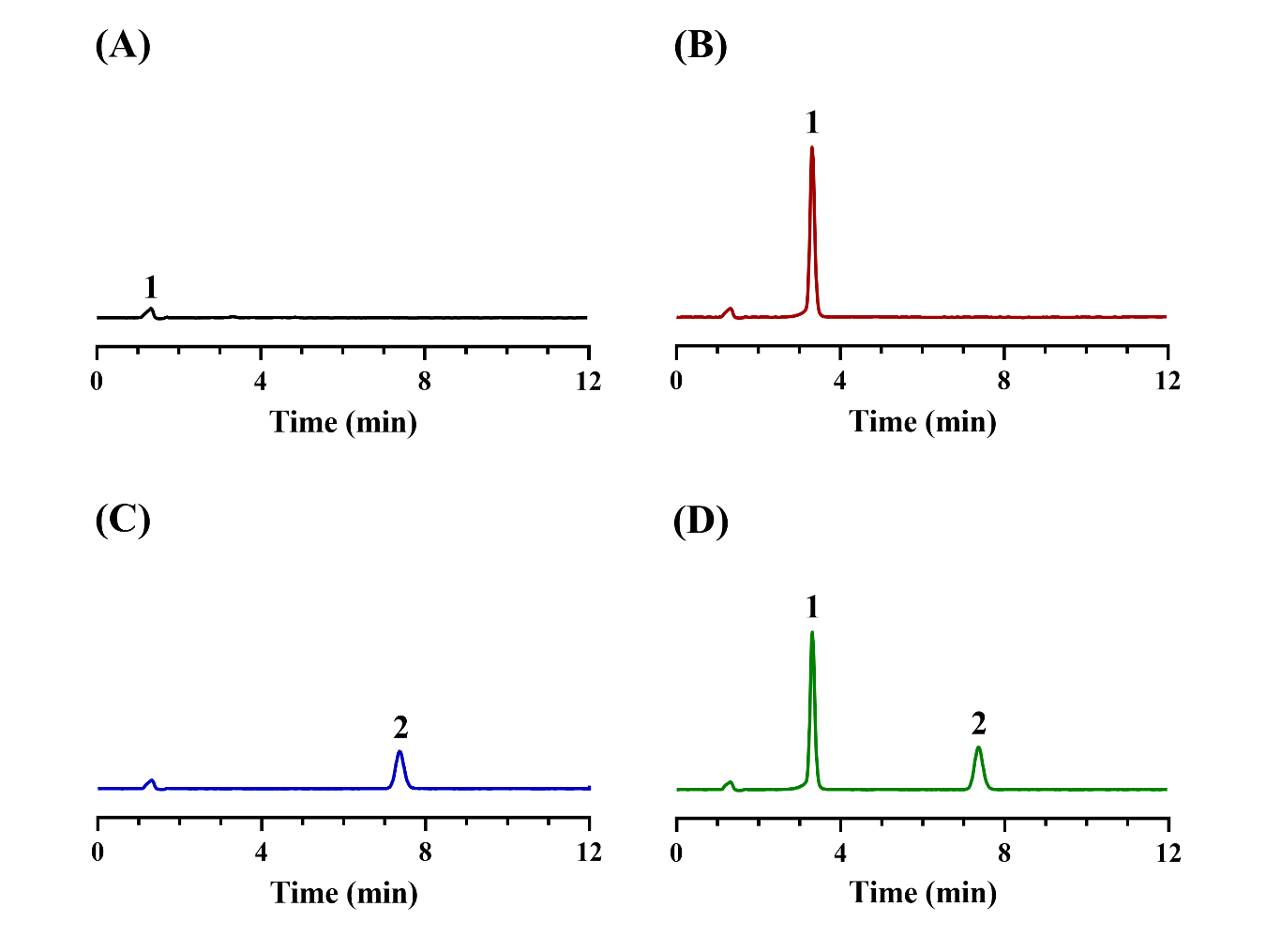


**Figure S5** Chromatograms of (A) blank mouse lung, (B) blank mouse lung with DOX solution, (C) blank mouse lung with DNR solution, and (D) blank mouse lung with DOX and DNR solution (Note: where 1 refers to blank mouse lung, 2 refers to DOX solution, 3 refers to DNR solution)


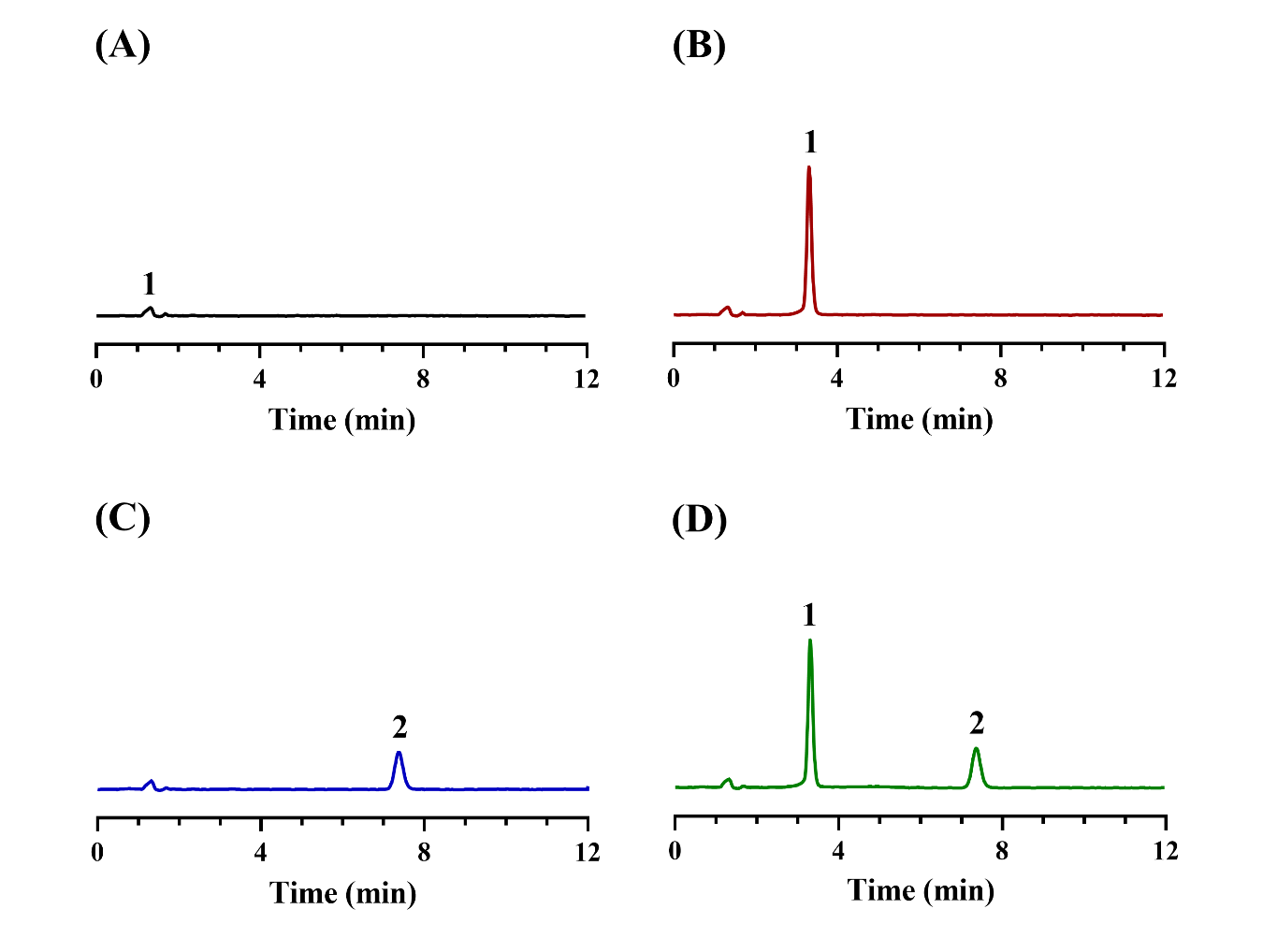


**Figure S6** Chromatograms of (A) blank mouse kidney, (B) blank mouse kidney with DOX solution, (C) blank mouse kidney with DNR solution, and (D) blank mouse kidney with DOX and DNR solution (Note: where 1 refers to blank mouse kidney, 2 refers to DOX solution, 3 refers to DNR solution)


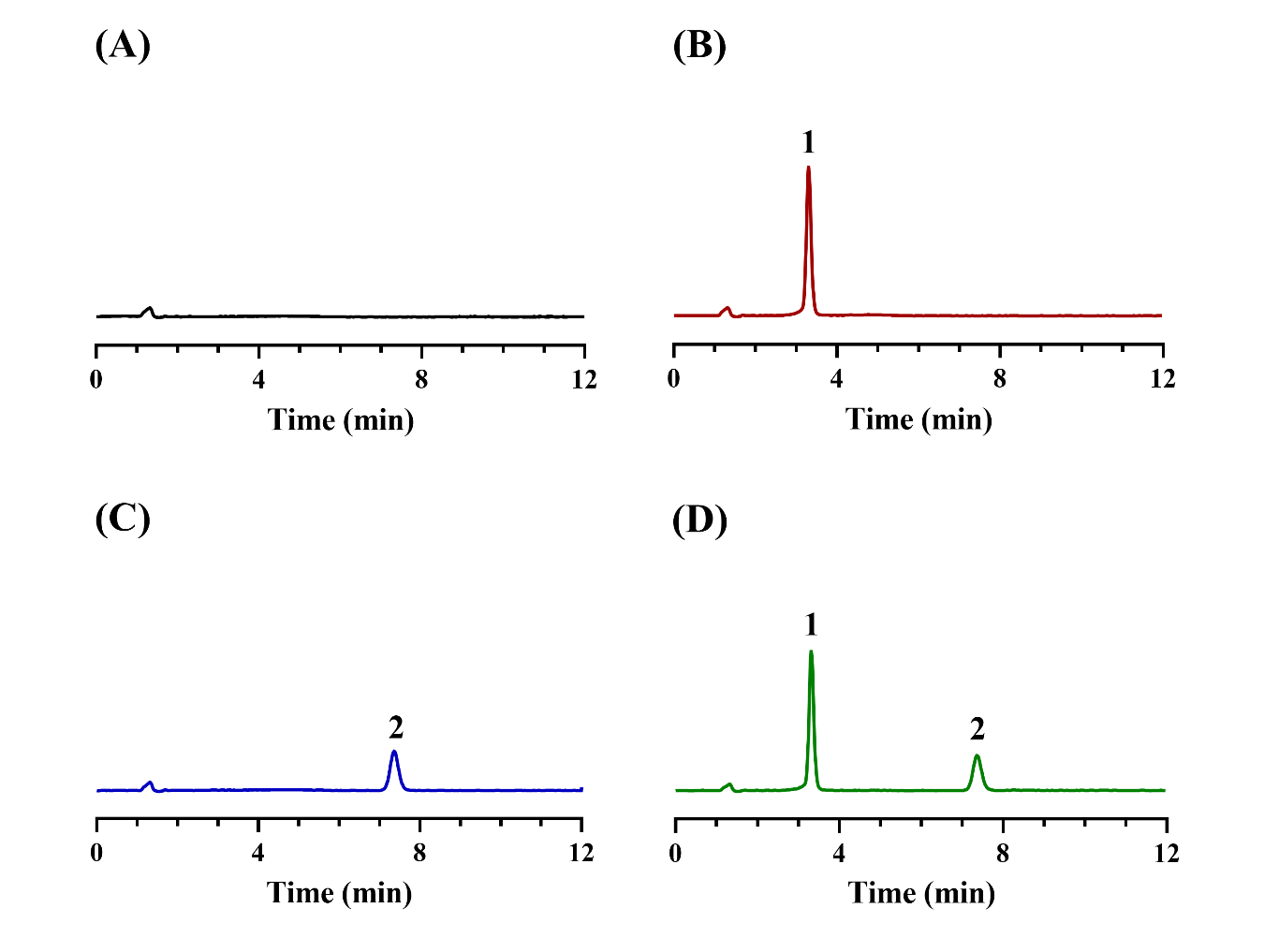


**Figure S7** Chromatograms of (A) blank mouse tumor, (B) blank mouse tumor with DOX solution, (C) blank mouse tumor with DNR solution, and (D) blank mouse tumor with DOX and DNR solution (Note: where 1 refers to blank mouse tumor, 2 refers to DOX solution, 3 refers to DNR solution)

## Supplementary Tables

**Table S1 Standard curve of DOX in plasma of rats and organs of mice**

| Samples | Regression equation | r |
| --- | --- | --- |
| Plasma | Y=0.3789X-0.03284 | 0.9998 |
| Heart | Y=0.5160X+0.01950 | 0.9979 |
| Liver | Y=0.5605X-0.04240 | 0.9997 |
| Spleen | Y=0.5066X-0.07610 | 0.9992 |
| Lung | Y=0.4977X-0.04410 | 0.9994 |
| Kidney | Y=0.5024X-0.07530 | 0.9993 |
| Tumor | Y=0.5098X-0.06090 | 0.9995 |

**Table S2 The test results of recovery of DOX in plasma of rats and organs of mice*,*** **n=6**

| Samples | 0.05 µg/mL | |  | 5 µg/mL | |  | 20 µg/mL | |
| --- | --- | --- | --- | --- | --- | --- | --- | --- |
|  | $\bar{x}$± s （µg/mL） | RSD（%） |  | $\bar{x}$± s  （µg/mL） | RSD（%） |  | $\bar{x}$± s  （µg/mL） | RSD（%） |
| Plasma | 82.31±3.86 | 4.69 |  | 88.34±2.03 | 2.30 |  | 97.41±1.04 | 1.10 |
| Heart | 85.75±3.81 | 4.44 |  | 88.55±2.73 | 3.08 |  | 96.84±1.65 | 1.71 |
| Liver | 83.96±3.13 | 3.73 |  | 88.65±1.95 | 2.20 |  | 95.41±2.38 | 2.49 |
| Spleen | 85.10±3.45 | 4.05 |  | 87.92±1.84 | 2.09 |  | 95.12±1.00 | 1.05 |
| Lung | 85.61±4.05 | 4.73 |  | 89.67±2.79 | 3.11 |  | 93.55±2.49 | 2.66 |
| Kidney | 84.12±2.26 | 2.69 |  | 91.54±2.53 | 2.77 |  | 95.24±1.88 | 1.97 |
| Tumor | 85.19±3.98 | 4.68 |  | 90.12±3.17 | 3.51 |  | 96.06±1.22 | 1.27 |

**Table S3 The test results of inter-day precision of DOX in plasma of rats and organs of mice, n=6**

| Samples | 0.05 µg/mL | |  | 5 µg/mL | |  | 20 µg/mL | |
| --- | --- | --- | --- | --- | --- | --- | --- | --- |
|  | Peak area ratio | RSD（%） |  | Peak area ratio | RSD（%） |  | Peak area ratio | RSD（%） |
| Plasma | 0.029±0.00077 | 2.60 |  | 2.22±0.0099 | 0.44 |  | 9.39±0.11 | 0.011 |
| Heart | 0.027±0.00094 | 3.37 |  | 2.86±0.026 | 0.92 |  | 11.51±0.35 | 3.11 |
| Liver | 0.029±0.0011 | 3.61 |  | 2.92±0.021 | 0.71 |  | 11.54±0.029 | 0.25 |
| Spleen | 0.029±0.0011 | 3.62 |  | 2.84±0.032 | 1.13 |  | 11.03±0.13 | 1.14 |
| Lung | 0.031±0.0080 | 2.57 |  | 2.92±0.019 | 0.66 |  | 10.95±0.088 | 0.80 |
| Kidney | 0.029±0.00044 | 1.52 |  | 2.81±0.030 | 1.06 |  | 11.08±0.12 | 1.08 |
| Tumor | 0.031±0.00092 | 2.93 |  | 2.95±0.061 | 2.07 |  | 11.40±0.19 | 1.67 |

**Table S4 The test results of intra-day precision of DOX in plasma of rats and organs of mice, n=6**

| Samples | 0.05 µg/mL | |  | 5 µg/mL | |  | 20 µg/mL | |
| --- | --- | --- | --- | --- | --- | --- | --- | --- |
|  | Peak area ratio | RSD（%） |  | Peak area ratio | RSD（%） |  | Peak area ratio | RSD（%） |
| Plasma | 0.030±0.00079 | 2.61 |  | 2.26±0.034 | 1.48 |  | 9.47±0.13 | 1.36 |
| Heart | 0.029±0.0011 | 3.89 |  | 2.91±0.034 | 1.18 |  | 11.22±0.23 | 2.02 |
| Liver | 0.029±0.00099 | 3.39 |  | 2.93±0.023 | 0.79 |  | 11.69±0.073 | 0.62 |
| Spleen | 0.029±0.0010 | 3.68 |  | 2.87±0.053 | 1.86 |  | 11.06±0.14 | 1.23 |
| Lung | 0.029±0.00056 | 1.91 |  | 2.89±0.029 | 1.00 |  | 10.81±0.13 | 1.20 |
| Kidney | 0.029±0.00062 | 2.12 |  | 2.77±0.035 | 1.28 |  | 11.12±0.059 | 0.53 |
| Tumor | 0.030±0.00098 | 3.21 |  | 2.95±0.077 | 2.61 |  | 10.97±0.23 | 2.05 |

**Table S5 The test results of accuracy of DOX in plasma of rats and organs of mice, n=6**

| Samples | 0.05 µg/mL | |  | 5 µg/mL | |  | 20 µg/mL | |
| --- | --- | --- | --- | --- | --- | --- | --- | --- |
|  | accuracy（%） | RSD（%） |  | accuracy（%） | RSD（%） |  | accuracy（%） | RSD（%） |
| Plasma | 97.69±2.54 | 2.60 |  | 96.49±0.42 | 0.43 |  | 100.22±1.21 | 1.21 |
| Heart | 101.19±3.28 | 3.24 |  | 99.86±0.92 | 0.92 |  | 100.02±3.11 | 3.11 |
| Liver | 94.21±3.74 | 3.97 |  | 100.76±0.73 | 0.72 |  | 99.96±0.25 | 0.25 |
| Spleen | 90.31±2.15 | 2.38 |  | 100.61±0.78 | 0.77 |  | 105.00±0.37 | 0.26 |
| Lung | 93.67±2.92 | 3.11 |  | 104.94±0.71 | 0.68 |  | 99.77±0.81 | 0.81 |
| Kidney | 95.39±1.58 | 1.65 |  | 100.88±1.08 | 1.07 |  | 99.95±1.08 | 1.08 |
| Tumor | 99.90±1.67 | 1.67 |  | 101.73±2.16 | 2.12 |  | 104.86±3.32 | 3.17 |

.
